# Cerebrovascular regulation during heat stress

**DOI:** 10.1113/EP093324

**Published:** 2026-03-13

**Authors:** Kanoko Ito, Manabu Shibasaki

**Affiliations:** ^1^ Endowed laboratory from the Sumitomo Electric Group CSR Foundation Nara Women's University Nara Japan; ^2^ Faculty of Engineering Nara Women's University Nara Japan

**Keywords:** cerebral perfusion, heat stress, hyperthermia

## Abstract

Given that the brain is a highly metabolic organ and is enclosed by the skull, effective regulation of cerebral blood flow (CBF), not only for oxygen and nutrients supply but also as a means of convective heat exchange, is critical for preventing excessive elevations in brain temperature. Exposure to a hot environment can increase brain temperature while also decreasing CBF. This reduction in CBF may decrease heat removal from the brain and further increases its susceptibility to overheating. Therefore, understanding the impact of heat stress on CBF regulation, and its underlying mechanisms, is crucial for developing appropriate countermeasures and preventive strategies against heat‐related injuries and mortality. Because hyperthermia profoundly complicates central circulatory regulation via extensive cutaneous vasodilation and blood volume distribution, CBF cannot be interpreted in isolation from systemic cardiovascular adjustments, particularly the preferential diversion of cardiac output toward the skin and extracranial tissues and the associated reduction in intracranial perfusion. Consequently, quantitative and region‐specific assessments of cerebral perfusion are required, as commonly used velocity‐based measurements may not fully capture changes in flow magnitude or differential responses between anterior and posterior cerebral circulations. Finally, since various brain functions are localized, it is essential to address regional differences in cerebral perfusion during stressors such as heat stress. The purpose of this review is to describe the key factors involved in the regulation of CBF and summarize how CBF is modulated under heat stress, with a focus on both quantitative changes and regional variability.

## Abstract (highlights)

- **What is the topic of this review?**
  This review focuses on changes in resting cerebral blood flow and cerebrovascular regulation, from both quantitative and regional perspectives, during passive heat stress.
- **What advances does it highlight?**
  This review highlights several key advances in our understanding of cerebrovascular regulation under heat stress, including observations of both quantitative and regional changes in cerebral blood flow, as well as an integrative discussion of how heat stress‐induced cardiovascular and cerebrovascular alterations contribute to orthostatic intolerance.

## 1 INTRODUCTION

Excessive heat stress poses health risks, such as heatstroke, leading to increased human morbidity and mortality (Ebi et al., 2021). Recent epidemiological studies suggest that extreme heat increases the risk of cardiovascular mortality, such as stroke (Singh et al., 2024; Zhu et al., 2024). As global warming progresses, the frequency, intensity and duration of heatwaves, with accompanying hotter summers, will increase (Perkins‐Kirkpatrick & Lewis, 2020). Based on recent climate change trends, future health risks from heat stress are predicted to become even more severe (Lüthi et al., 2023). To prevent illness and death caused by heat stress, it is necessary to clarify its physiological mechanisms and propose appropriate countermeasures.

Early symptoms of heat‐related illness include dizziness and lightheadedness, palpitations and tachycardia, fatigue and malaise, profuse sweating, and facial flushing or pallor. In more advanced heat exhaustion, additional symptoms may appear, such as nausea and vomiting, headache, muscle cramps or spasms, weakness and impaired concentration, as well as a sensation of cold, clammy skin. Some of the adverse health effects caused by heat stress may be due to changes in cerebral circulation (Yoneda et al., 2024). Since the brain has a high metabolic rate and therefore requires continuous blood flow to supply oxygen and nutrients, facilitate the removal of metabolic by‐products and maintain acid–base balance, while also removing heat from brain tissue through convective heat transfer, maintaining cerebral perfusion is important. Exposure to a hot environment alters various regulatory functions. In this review, we briefly summarize cardiovascular responses during heat stress and then focus on how cerebral perfusion is regulated during hyperthermia. We discuss the static shift in resting cerebral blood flow (CBF), as well as the dynamic modifications in cerebrovascular regulatory responses to perturbations, such as changes in arterial CO_2_ pressure or blood pressure.

## 2 CARDIOVASCULAR RESPONSES UNDER HEAT STRESS

Passive heat stress causes myriad physiological responses. Regarding cardiovascular responses to heat stress, these adjustments reflect not only marked changes in cardiac function but also profound alterations in vascular tone across multiple vascular beds, particularly those involved in thermoregulation and blood volume distribution. A well‐known figure from Rowell's review (1974) ‘Human cardiovascular adjustment to exercise and thermal stress’ is often cited. Notably, despite the absence of exercise and with subjects lying supine at rest, cardiac output nearly doubled compared to resting values (Figure 1). Approximately half of the elevated cardiac output is calculated to be directed to the skin, reflecting the unique cholinergic active vasodilator system in human cutaneous circulation (Mueller et al., 2017; Shibasaki et al., 2006, 2007). Based on the review by Crandall & Wilson (2015) and including findings from more recent studies, we briefly summarize cardiovascular responses during heat stress.

**FIGURE 1:**
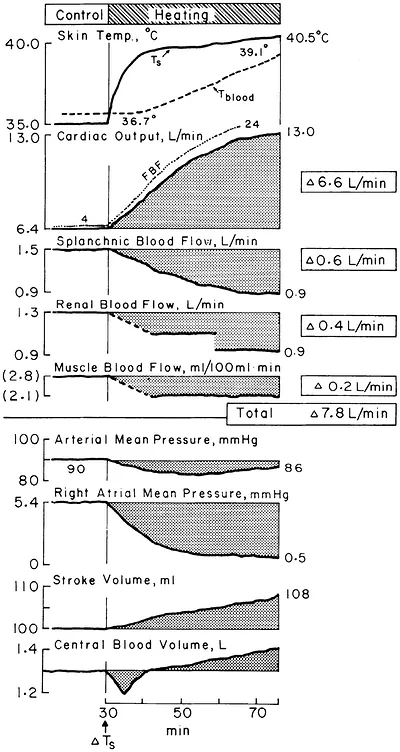
Typical cardiovascular responses to whole‐body passive heat stress in humans. Reproduced from Rowell (1974), with permission.

Cardiac output is primarily regulated by heart rate and stroke volume. Heart rate intrinsically rises by approximately 7 bpm for every 1.0°C increase in core temperature (*T*_core_), even when autonomic control is blocked. Heating the myocardium directly increases the slope of Phase IV of the cardiac action potential and shortens the action potential duration of the sinoatrial and atrioventricular nodes. In addition, other cardiac myocytes, including Purkinje fibres, atrial myocytes and ventricular myocytes, also exhibit a shortened action potential duration. Beyond these direct effects, elevated temperature enhances the conduction velocity of pacemaker signals to adjacent cardiac myocytes via its influence on gap junctions. The activity of sinoatrial and atrioventricular nodes is further modulated by sympathetic and parasympathetic input. Stroke volume, unlike heart rate, typically remains relatively stable during passive heat stress in supine individuals, although a slight decrease may occur depending on the intensity of the thermal load. Stroke volume is influenced by four key factors: preload, afterload, contractility and diastolic function. These factors interact intricately to support the elevation in cardiac output and maintenance of arterial blood pressure despite a marked reduction in systemic vascular resistance and possibly circulating blood volume.

‘Cardiac preload’ is defined as the tension generated by ventricular myocyte stretching during end‐diastole and is a key determinant of stroke volume through the Frank–Starling mechanism. Because quantifying changes in myocyte length in vivo remains challenging, the relation between preload and force production is usually assessed using surrogate measures of ventricular preload, such as right atrial pressure, central venous pressure, pulmonary capillary wedge pressure, and left‐ventricular end‐diastolic volume (Crandall & Wilson, 2015). Each index has advantages and limitations in reflecting left ventricular preload and associated physiological changes. The mechanisms underlying heat‐induced reductions in preload are multifactorial and are largely driven by vascular adjustments, including extensive cutaneous vasodilation and redistribution of blood volume away from the central circulation. Increased sweating reduces plasma and interstitial fluid volumes (Sawka et al., 2005), while regional shifts in blood volume – especially large increases in cutaneous vascular volume for heat dissipation – contribute to decreases in central blood volume and associated reductions in ventricular filling pressures (Crandall & Wilson, 2015). Elevated heart rates that occur with heat stress shorten diastolic filling time, which has the potential to further reduce preload on a per‐beat basis.

‘Cardiac afterload’ is influenced by peripheral vascular resistance, arterial compliance, arterial segmental constriction and blood viscosity. During heat stress, total peripheral resistance decreases with an increase in *T*_core_ (Rowell, 1974), reflecting widespread vasodilation, particularly within the cutaneous circulation. On the other hand, arterial properties, including stiffness of both central and peripheral arteries, are unchanged by heat stress (Ganio et al., 2011). Despite reductions in ventricular filling pressure during heat stress, stroke volume can be maintained through compensatory cardiac mechanisms. In the context, the leftward and upward shift in the Frank–Starling relationship in heated humans allows for stroke volume to be maintained despite a reduction in ventricular filling pressure. Nevertheless, because multiple factors can affect cardiac afterload under passive heat stress, further studies are needed to fully understand the relative contributions of afterload versus other factors influencing the Frank–Starling relationship in heat stressed humans.

‘Cardiac contractility’ increases during heat stress (Crandall & Wilson, 2015; Shibasaki et al., 2015). Multiple imaging studies (e.g., radionucleotide multi‐gated acquisition and echocardiography‐derived increases in ejection fraction), isovolumic acceleration of the septal and lateral mitral annulus, and increases in left‐ventricular twist rates (Nelson et al., 2010; Stöhr et al., 2011) all indicate positive inotropic effects of passive heat stress. Shibasaki et al. (2015) demonstrated that left atrial and ventricular systolic function increased in able‑bodied individuals during heat stress, whereas this heat‐induced augmentation was attenuated in paraplegics and was absent in tetraplegics. These findings suggest that cardiac sympathetic innervation is essential for normal left ventricular systolic responses to heat stress. Taken together, the increase in left ventricular systolic function during heat stress is mainly driven by elevated *T*_core_, with possible contribution from skin thermoreceptor stimulation, in causing increases in neural activity to the cardiac tissue resulting in increases in indices of cardiac contractility.

‘Diastolic function’ encompasses both ventricular relaxation and filling. Indices of left‐ventricular compliance, such as volume‐to‐filling pressure ratios, remain unchanged during passive heating. Although most diastolic indices are preload‐dependent and typically decline with reductions in central venous pressure, pulmonary capillary wedge pressure, end‐diastolic volume, and/or central blood volume, indices of diastolic function are preserved under passive heat stress. Brothers et al. (2014) demonstrated that restoring central venous pressure, which is reduced during heat exposure, to pre‐heating levels via volume infusion enhanced diastolic indices that were otherwise unchanged in normothermic and heat‐stressed conditions. Nelson et al. (2010) proposed that enhanced left‐ventricular untwisting preserves the forces driving early filling during passive heat stress. Mechanical factors, including increased recoil and ventricular suction, together with lusitropy – the rate of cardiac myocyte relaxation – appear to support diastolic function under heat stress (Stöhr et al., 2011). In addition, the preserved diastolic function in patients with tetraplegia suggests that sympathetic influence is minimal (Shibasaki et al., 2015).

Finally, the increased cardiac output induced by heat stress is primarily directed to the cutaneous circulation as a result of pronounced cutaneous vasodilation, while blood flow to the splanchnic and renal regions is reduced (Crandall & Wilson, 2015). These vascular adjustments are essential for heat dissipation but simultaneously complicate the maintenance of central blood volume and arterial pressure. Blood supplied to the brain flows into the cranial cavity via the internal carotid and vertebral arteries (ICA and VA), which arise from the common carotid and subclavian arteries, respectively. Ogoh et al. (2013) estimated blood flow toward the head during heat stress by measuring flow through the ICA, external carotid artery (ECA) and VA. Using these approaches, the total blood flow to the head increased in proportion to cardiac output during heat stress (Figure 2). However, this increase was largely dependent on increases in ECA blood flow to the skin, while blood flow through the ICA and VA, which supply intracranial regions, decreased as body temperature rose. The cerebrovasculature is regulated differently from other vascular beds, and the observed reductions appear to be influenced not only by cardiovascular adjustments but also by respiratory alterations induced by hyperthermia, which are discussed in more detail in the following sections.

**FIGURE 2:**
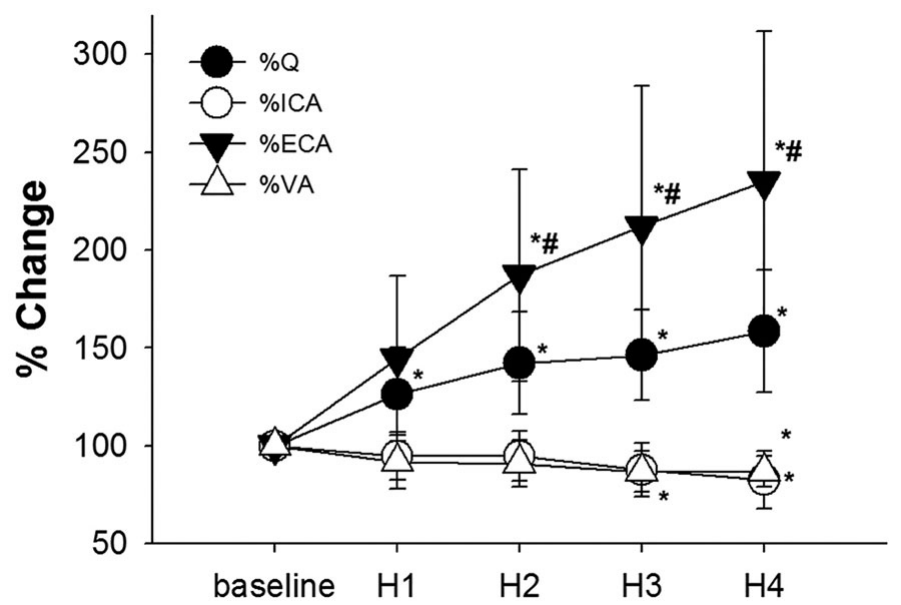
The percentage of changes in the cardiac output (*Q*), internal carotid artery (ICA), external carotid artery (ECA), and vertebral artery (VA) blood flow from normothermia (baseline) throughout passive heat stress. Values are means ± SD. *P < 0.05 different from the baseline; ^#^ *P *< 0.05 different from H1. H, heat. Reproduced from Ogoh et al. (2013), with permission.

## 3 STATIC CHANGES IN CBF UNDER HEAT STRESS

### 3.1 Heat stress and regional CBF

The human brain constitutes only 2–3% of total body mass, yet accounts for approximately 20% of resting whole‐body oxygen consumption, which necessitates precise regulation of blood flow and consequently oxygen delivery for optimal function. At an average CBF of ∼700 mL/min and a constant cerebral metabolic rate of oxygen ($\mathrm{CM}R_{O_{2}}$) of ∼50 mL/min, cerebral heat balance is maintained with a cerebral arterial‐to‐venous blood temperature gradient of ∼0.3–0.5°C (Bain et al., 2015). The ICA, a major branch arising from the common carotid artery alongside the ECA, supplies ∼70% of total CBF. The bilateral VAs merge to form the basilar artery, which distributes ∼30% of CBF primarily to the brainstem, cerebellum, and occipital lobes. The ICA predominantly supplies the middle cerebral artery (MCA), whereas the VA primarily contributes to the posterior cerebral artery (PCA). The MCA provides blood to a larger total brain mass, principally the frontal and temporal cortices and anterior portions of the occipital cortex, whereas the PCA supplies a substantially smaller blood flow to the posterior circulation.

In human studies, CBF is assessed using transcranial Doppler ultrasound (TCD), vascular ultrasound, or magnetic resonance imaging (MRI) techniques such as blood oxygen level‐dependent (BOLD) imaging and arterial spin labelling (ASL). TCD enables measurement of blood velocity of large intracranial cerebral arteries, such as the MCA and PCA, but it does not provide an arterial diameter, which results in limitations in the interpretation of flow in these arteries. With vascular ultrasound, blood flow can be calculated by measuring arterial diameter and blood velocity of extracranial cerebral conduit arteries, such as the ICA and VA. MRI offers higher spatial resolution compared to ultrasound‐based techniques; however, its temporal resolution is comparatively limited at present, ranging from hundreds of milliseconds to a few seconds.

Since the late 1990s, following the work of Doering et al. (1996), many studies have assessed the effects of heat stress on cerebral perfusion, initially measuring MCA mean velocity (*V*_mean_) via TCD as a surrogate for global CBF. The majority of these studies employed supine heating using a water‐perfused suit, and have demonstrated that CBF decreases by approximately 10–15% for each 1°C increase in *T*_core_, although the physiological responses vary with different heating methods and posture (Bain et al., 2015; Gibbons et al., 2021). These investigations have consistently reported reductions in both MCAV_mean_ and PCAV_mean_ during passive whole‐body heating when the heat stress is sufficiently pronounced. Quantitatively, Sato & Sadamoto (2010) were the first to report regional differences in blood flow through the internal carotid (${\dot{Q}}_{\mathrm{ICA}}$) and vertebral (${\dot{Q}}_{\mathrm{VA}}$) arteries during exercise‐induced increases in body temperature. In a subsequent study, the same group passively increased *T*_core_ by +1.4°C using a water‐perfused suit and reported that blood flow in the ICA and VA decreased by approximately 15% (Ogoh et al., 2013). In contrast, around the same time, Bain et al. (2013) reported that during severe passive hyperthermia (+2°C *T*_core_), ${\dot{Q}}_{\mathrm{VA}}$ (∼30%) decreased more than ${\dot{Q}}_{\mathrm{ICA}}$ (∼20%). However, the difference in reductions between simultaneously measured MCAV_mean_ and PCAV_mean_ was not statistically significant (23% vs. 18%, respectively). Nelson et al. (2011) also demonstrated that MCAV_mean_ (26%) and PCAV_mean_ (23%) were similarly reduced under a +1.8°C rise in *T*_core_. Collectively, these findings indicate that while both anterior and posterior CBF decrease during passive heat stress, regional differences are not universally observed.

Recent studies utilizing MRI to assess the regional distribution of CBF support the hypothesis that heat stress induces spatial heterogeneity in cerebral perfusion. Qian et al. (2014) evaluated regional blood flow variation under mild heat stress (+1°C *T*_core_) using MRI with ASL. Although mean CBF in the whole brain, grey matter, and white matter tended to decrease following heat exposure, these changes did not reach statistical significance. Nevertheless, localized redistribution of blood flow was observed, primarily involving the prefrontal cortex, somatosensory cortex, and limbic regions. Tan et al. (2024) investigated cerebral perfusion via MRI under severe heat stress (+2.5°C *T*_core_) and reported a significant reduction in global grey matter perfusion, along with decreased regional perfusion in the precentral and postcentral gyri.

### 3.2 Mechanisms of CBF alterations under heat stress

Although the mechanism(s) by which heat stress reduces cerebral perfusion are multifactorial and not readily apparent, these responses are closely related to changes in systemic circulation and breathing patterns caused by an increase in body temperature. The regulation of CBF is a dynamic process that responds to fluctuations in perfusion pressure, cerebral metabolic demand, humoral influences, autonomic nervous system activity and arterial CO_2_ partial pressure (Ainslie & Duffin, 2009). As described in section 2, an elevation in core temperature redistributes systemic blood flow and modulates cardiac output, thereby influencing cerebral perfusion pressure. In addition, a rise in core temperature can increase minute ventilation (${\dot{V}}_{E}$) (hyperthermic hyperventilation), regardless of physical activity (Tsuji et al., 2016). Given that oxygen uptake remains largely unchanged from normothermic levels in passively heated resting subjects, the influence of metabolic factors on hyperthermic hyperventilation appears to be trivial. Consequently, this hyperventilation results in excessive elimination of CO_2_ from the body, decreasing arterial CO_2_ partial pressure ($P_{\mathrm{aC}O_{2}}$) (hypocapnia). The cerebrovasculature is highly sensitive to changes in the $P_{\mathrm{aC}O_{2}}$, with CBF decreasing by 1–3% for each 1 mmHg fall in $P_{\mathrm{aC}O_{2}}$ from eucapnia in normothermia (Bain et al., 2015). Though highly variable, a 1.5–2.0°C increase in *T*_core_ from passive heating typically reduces $P_{\mathrm{aC}O_{2}}$ by ∼5–15 mmHg and decreases CBF by ∼20% (Bain et al., 2014).

Several studies have investigated the contribution of $P_{\mathrm{aC}O_{2}}$ reduction to the heat‑induced reduction in CBF by restoring $P_{\mathrm{ETC}O_{2}}$ to normothermic levels by hypercapnic inhalation during heat stress. However, the CBF recovery rates (increase in CBF due to $P_{\mathrm{ETC}O_{2}}$ restoration/CBF reduction induced by heat stress), which reflect the contribution of CO_2_, vary considerably across experiments. Fujii et al. (2008) and Tsuji et al. (2018) investigated the CBF recovery rate during heat stress induced by lower‑leg hot‑water immersion combined with a water‑perfused suit in the seated position. They reported that the recovery rates for MCAV_mean_ were 28%, 38%, and 37% at *T*_core_ elevations of +1.2°C, +1.6°C and +2.0°C, respectively. In contrast, other experiments applying heat stress in the supine position using a water‑perfused suit have demonstrated a greater contribution of $P_{\mathrm{aC}O_{2}}$. Brothers et al. (2009a) used a computer‐controlled end‐tidal gas targeting system to return $P_{\mathrm{ETC}O_{2}}$ to normothermic levels in heat‐stressed subjects (+1.4°C *T*_core_) and found that approximately 50% of the decrease in cerebral perfusion is $P_{\mathrm{aC}O_{2}}$ dependent. Following that, a higher proportion of $P_{\mathrm{ETC}O_{2}}$ contribution has been reported under severe heat stress of +1.8–2.0°C *T*_core_. Nelson et al. (2011) reported that under +1.8°C *T*_core_, the CBF recovery rates were 67% for MCAV_mean_ and 85% for PCAV_mean_. Bain et al. (2013) reported that under +2.0°C *T*_core_, the CBF recovery rates were 79%, 114%, 75% and 90%, for ${\dot{Q}}_{\mathrm{ICA}}$, ${\dot{Q}}_{\mathrm{VA}}$, MCAV_mean_ and PCAV_mean_, respectively, indicating that the recovery rate was higher for posterior circulation. As discussed in detail in Section 4.1, CBF‐CO_2_ reactivity has been shown to be largely unaffected by heat stress. Therefore, the variability in the reported contributions of $P_{\mathrm{aC}O_{2}}$ may be attributable to hydrostatic effects due to posture and the severity of dehydration induced by heat stress (Lucas et al., 2018; Ota et al., 2019).

Additional possible mechanisms responsible for a reduction in CBF during hyperthermia have been proposed, such as increased cerebral sympathetic nervous stimulation (Brothers et al., 2009b) and thus reduction in cerebral vascular conductance (Wilson et al., 2006), decreased cerebral perfusion pressure due to decreased central arterial blood pressure (Bain et al., 2014), and redistribution of cardiac output (Ogoh et al., 2013; Sato et al., 2016). Although these contributions remain unclear, they are suggested to vary depending on factors such as the presence or absence of exercise, the severity of heat stress, and the cerebral region. During exercise in hot versus thermoneutral environments, the reduction in ICA blood flow was associated with a decrease in $P_{\mathrm{ETC}O_{2}}$, whereas changes in VA blood flow were linked to alterations in cardiac output (Sato et al., 2016). Without exercise, changes in MCAV_mean_ and PCAV_mean_ were primarily correlated with stroke volume and cardiac preload under mild heat stress (+0.8°C *T*_core_), with no significant correlation with cardiac output. In contrast, under severe heat stress (+1.8°C *T*_core_), changes in MCAV_mean_ and PCAV_mean_ were strongly correlated with $P_{\mathrm{ETC}O_{2}}$ (Nelson et al., 2011).

Furthermore, the reduction in CBF induced by hyperthermia may be partially offset by increased cerebral metabolism, which enhances blood flow via neurovascular coupling. Recently, Bain et al. (2020) demonstrated that hyperthermia independently elevates cerebral metabolic activity and consequently augments CBF, which was observed upon eliminating the confounding effects of respiratory alkalosis via controlled CO_2_ inhalation. Specifically, $\mathrm{CM}R_{O_{2}}$ increased by ∼20% during hyperthermia (+2.0°C *T*_core_). This pronounced increase in cerebral perfusion within the posterior region, likely driven by local metabolic demands, may contribute to the regional differences in CBF observed under heat stress.

## 4 DYNAMIC CEREBROVASCULAR REGULATION UNDER HEAT STRESS

### 4.1 Heat stress and CO_2_ reactivity

Heat stress not only shifts resting CBF but also modifies the regulatory responses to fluctuations in arterial CO_2_ partial pressure and blood pressure. The CBF‐CO_2_ reactivity (i.e., changes in CBF for a given change in $P_{\mathrm{aC}O_{2}}$) is reported to increase during exercise and this increase is further enhanced under heat stress (Rasmussen et al., 2006); however, the effect of passive heat stress on CBF‐CO_2_ reactivity at rest appears to be relatively small compared to that during exercise. The majority of studies concluded that even a +2°C increase in *T*_core_ does not alter CBF‐CO_2_ reactivity, even when combined with mild orthostatic stress or dehydration (Bain et al., 2015), while a few studies reported an increase in CBF‐CO_2_ reactivity in the relatively limited range of $P_{\mathrm{ETC}O_{2}}$ (Gibbons et al., 2020; Lucas et al., 2008). In contrast to the previous reports that examined CBF‐CO_2_ reactivity only in intra‐cranial arteries (i.e., MCA and PCA), simultaneous measurements of extra‐cranial arteries suggested that the extent to which heat stress affects CBF‐CO_2_ reactivity varies across vascular regions. Ogoh et al. (2014) demonstrated that a +1.5°C increase in *T*_core_ reduced CBF‐CO_2_ reactivity in the ICA, but had no effect on the VA and MCA reactivity. Notably, the absolute CO_2_ reactivity in the VA was lower than in the ICA, regardless of heat stress status. Although the mechanisms underlying regional differences in CBF‐CO_2_ reactivity remain unclear, the relatively stronger sympathetic innervation of the ICA compared to the VA (Koep et al., 2022) may explain the reduced CO_2_ reactivity observed under heat stress, possibly due to heat‐induced sympathetic vasoconstriction.

Recent studies have assessed cerebrovascular‐CO_2_ reactivity using cerebrovascular conductance (CBVC) or its inverse, resistance, to isolate the influence of blood pressure on CBF‐CO_2_ reactivity (Low et al., 2008; Skinner et al., 2024). These studies suggest that CBVC‐CO_2_ reactivity is minimally affected by heat stress, although a slight increase may occur during hypocapnia. Low et al. (2008) reported that during heat stress sufficient to increase *T*_core_ by 1.1°C, CBVC‐CO_2_ reactivity during hypocapnia slightly increased, although the changes were not statistically significant. Skinner et al. (2024) reported that CBF‐CO_2_ reactivity to hypocapnia was unaffected by heat stress of +1.3°C *T*_core_, whereas CBVC‐CO_2_ reactivity tended to increase. In contrast, in that study CBF‐CO_2_ reactivity to hypercapnia decreased under heat stress, while CBVC‐CO_2_ reactivity remained unchanged, suggesting that this discrepancy may be attributable to changes in mean arterial pressure. No regional differences in CBF‐ and CBVC‐CO_2_ reactivity were observed between the MCA and PCA during hyperthermia. However, given that hyperthermia alters the distribution of CBF, as mentioned above (Qian et al., 2014; Tan et al., 2024), and that regional differences in CBF‐CO_2_ reactivity exist even at normothermia (Fierstra et al., 2013), further studies using MRI or other techniques are warranted to address this question.

### 4.2 Heat stress and cerebral autoregulation

The maintenance of cerebral perfusion is critical for preserving cerebral function. The ability to maintain CBF by adjusting cerebrovascular resistance across a range of arterial pressures is called cerebral autoregulation (CA). CA is typically assessed by measuring CBF changes in response to steady‐state changes in arterial blood pressure (‘static’ CA) or transient blood pressure fluctuations (‘dynamic’ CA). Dynamic CA (dCA) is assessed using the thigh cuff technique or transfer function analysis (TFA) (Tzeng et al., 2012). The thigh cuff technique involves thigh‐cuff inflation followed by rapid cuff deflation, and recovery of CBF is used to quantify the dCA. TFA evaluates the frequency‐dependent relationship between spontaneous fluctuations in arterial blood pressure and CBF. The few studies that used the thigh cuff technique showed that dCA is improved under mild hyperthermia (+∼0.4°C *T*_core_) (Doering et al., 1999), whereas no difference in dCA was observed between normothermia and moderate hyperthermia (+∼1°C *T*_core_) (Low et al., 2009). On the other hand, the dCA assessed by TFA is either unchanged or partially improved with passive heat stress (Bain et al., 2015). However, a limitation of the studies employing TFA is that fluctuation of arterial blood pressure may be too small to elicit cerebrovascular autoregulation. Using large oscillations in arterial pressure imposed by cyclic lower body negative pressure (LBNP), Brothers et al. (2009b) found reduced transfer function gain in heat stressed individuals, which suggests improved dCA, but only in the low‐frequency range. Also, Taghizadeh et al. (2025) calculated a time‐adjusted ratio between MCAV_mean_ and MAP changes separately for increasing and decreasing MAP phases during oscillatory LBNP in heat stressed individuals. Their findings indicated an absence of directional sensitivity in the cerebral pressure–flow relationship during normothermia and hyperthermia (+∼1°C *T*_core_). Moreover, sensitivity metrics at very low frequency LBNP were reduced during hyperthermia compared to normothermia, suggesting that dCA may become more efficient under moderate heat stress.

### 4.3 Heat stress and orthostatic tolerance

Heat stress profoundly reduces orthostatic tolerance (Schlader et al., 2016). Dizziness and lightheadedness, which are among the early symptoms of heat‐related illness, are associated with this orthostatic intolerance. Imposing an orthostatic challenge using LBNP or head‐up tilt in heat‐stressed individuals causes a further reduction in arterial blood pressure and cerebral perfusion. When the central hypovolemic insult induced by an orthostatic challenge surpasses the capacity of sympathetic‐mediated cardiovascular regulation, cerebral perfusion can no longer be maintained, resulting in pre‐syncopal symptoms and ultimately syncope.

Although the mechanisms underlying the reduction in orthostatic tolerance during heat stress remain unclear, they are likely governed by multiple interacting factors (Schlader et al., 2016). A reduction in cerebral perfusion can result from decreased cerebrovascular conductance and/or reduced cerebral perfusion pressure. Previous studies suggest that the reduction in orthostatic tolerance during heat stress is attributable more to decreases in baseline cerebral perfusion pressure or its regulatory responses than to reductions in baseline cerebrovascular conductance or its regulatory responses.

Interestingly, the reduction in orthostatic tolerance induced by heat stress is independent of the decrease in baseline CBF resulting from decreased cerebrovascular conductance associated with hyperventilation‐induced hypocapnia (Lucas et al., 2013; Shibasaki et al., 2020). Lucas et al. (2013) reported that maintaining MCAV_mean_ via CO_2_ inhalation during heat stress (+1.3°C *T*_core_) did not improve orthostatic tolerance. Consistent with that finding, Shibasaki et al. (2020) demonstrated that even when whole‐brain blood flow measured from the ICA and VA were increased above normothermic baseline levels through CO_2_ inhalation during heat stress (+1.4°C *T*_core_), orthostatic tolerance was not improved (Figure 3). With respect to changes in the regulation of cerebrovascular conductance, as described in Sections 4.1 and 4.2, previous studies have shown that heat stress has little effect on CO_2_ reactivity, and that dynamic CA during orthostatic stress is either unchanged or even enhanced under heat‑stressed conditions.

**FIGURE 3:**
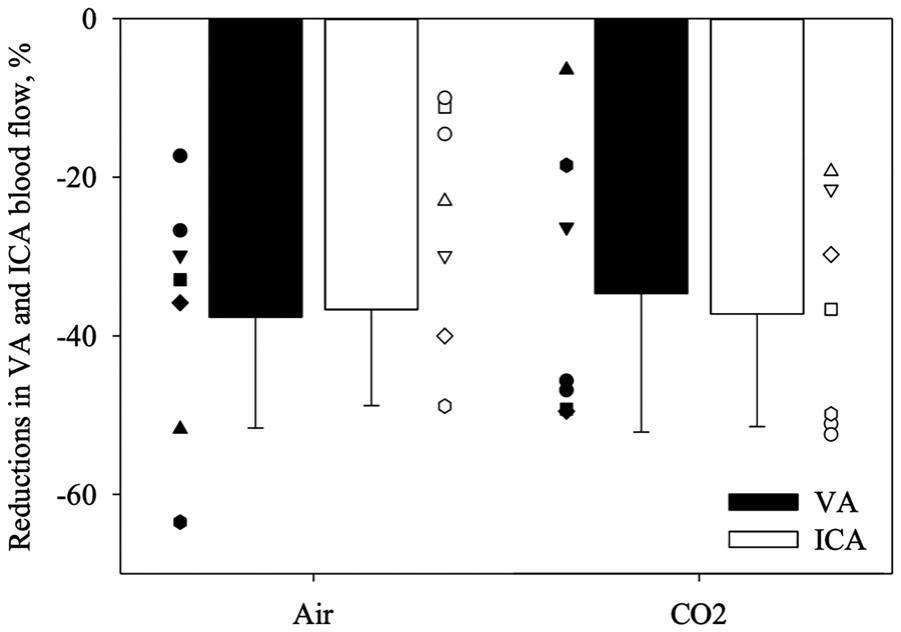
Relative changes in vertebral artery (VA, filled bar/symbols) and internal carotid artery (ICA, open bar/symbols) blood flows during lower‐body negative pressure while breathing room air (left dataset) or a hypercapnic gas mixture (right dataset). Note that despite the administration of hypercapnic gas, both ICA and VA blood flows similarly decreased from normothermic values at the end of lower‐body negative pressure while heat stress. Reproduced from Shibasaki et al. (2020), with permission.

On the other hand, numerous studies have provided evidence that reductions in central blood volume due to peripheral blood redistribution and dehydration (Keller et al., 2009; Wilson et al., 2002), as well as inadequate vasoconstriction to a hypotensive challenge (Ganio et al., 2012), impair blood pressure regulation and thereby compromise the maintenance of CBF. Wilson et al. (2002) demonstrated that whole‐body skin cooling improves orthostatic tolerance in heat‐stressed individuals, accompanied by the preservation of MCAV_mean_ and arterial blood pressure. Keller et al. (2009) reported that reduced orthostatic tolerance with heat stress can be entirely mitigated by restoring central venous pressure (and presumably central blood volume) with a rapid dextran infusion before an orthostatic challenge, although CBF was not obtained. Furthermore, it has been suggested that blood pressure regulation via systemic vascular conductance is impaired during heat stress. Ganio et al. (2012) conducted LBNP to pre‐syncope under heat stress of +1.2°C *T*_core_ and reported that systemic vascular conductance (calculated by cardiac output/mean arterial blood pressure) failed to decrease appropriately despite marked reductions in arterial pressure associated with pre‐syncope. Also, baroreflex responsiveness (Crandall et al., 2003) and the capacity to increase sympathetic activity during orthostatic stress (Cui et al., 2004; Gagnon et al., 2015) are largely unaffected by passive heat stress. One proposed mechanism for this attenuated vascular responsiveness during heat stress is that increases in nitric oxide, a potent vasodilator, suppress sympathetic‐mediated cutaneous vasoconstriction (Shibasaki et al., 2006, 2007), with similar responses being observed in the skeletal muscle vasculature (Ives et al., 2012). Together, these findings indicate that heat‐induced orthostatic intolerance does not arise from a single mechanism, but rather reflects the combined effects of reduced central blood volume (and likely ventricular filling pressures) and impaired blood pressure regulation via systemic vascular conductance during heat stress.

To mitigate heat‑induced orthostatic intolerance, several countermeasures have been evaluated. Whole‐body skin cooling (Wilson et al., 2002) and volume infusion (Keller et al., 2009) have been shown to be effective countermeasures. Although face cooling elevates blood pressure during orthostatic stress (Johnson et al., 2017), its effectiveness is limited under heat stress due to attenuated cutaneous vasoconstriction in response to this cooling stimulus (Schlader et al., 2018; Tourula et al., 2024). Moreover, normalizing arterial CO_2_ tension during heat stress not only fails to preserve orthostatic tolerance, but is also suggested to enhance the cerebral uptake of microvesicles carrying inflammatory cargo (Parikh et al., 2023). Additional research is warranted to better understand the mechanisms of heat‐induced orthostatic intolerance and establish effective interventions.

## 5 CONCLUSIONS

Whole‐body heat stress increases cardiac output and can increase blood supply to the head, with much of the increase in head blood flow being distributed to the extracranial vessels via the ECA. In contrast, intracranial blood flow is regulated independently of the increase in cardiac output, given well recognized decreases in ICA and VA blood flows while in this thermal condition. The primary mechanism(s) responsible for heat stress‐induced reductions of CBF are hypocapnia‐induced vasoconstriction likely due to hyperventilation, reductions in arterial pressure and/or central blood volume (when such reductions occur), and perhaps a redistribution of blood flow toward the face and away from cerebral tissues. In the intracranial arteries (i.e., MCA and PCA), both the degree of reduction in blood flow due to heat stress and the CO_2_ reactivity are similar, whereas these responses in the extracranial arteries (i.e., ICA and VA) differ somewhat. Given a host of factors that can influence both quantitative and regional changes in CBF in heat‐stressed humans, further detailed investigations may reveal aspects of CBF regulation under such conditions that are not currently detectable.

## AUTHOR CONTRIBUTIONS

Manabu Shibasaki was responsible for the concept of the review. Manabu Shibasaki and Kanoko Ito contributed to the design and writing of the manuscript. Both authors approved the final version of the manuscript and agree to be accountable for all aspects of the work, ensuring that questions related to the accuracy or integrity of any part of the work are appropriately investigated and resolved. All those who qualify for authorship are listed.

## CONFLICT OF INTEREST

None declared.
